# Targeting vulnerable groups of health poverty alleviation in rural China— what is the role of the New Rural Cooperative Medical Scheme for the middle age and elderly population?

**DOI:** 10.1186/s12939-020-01236-x

**Published:** 2020-09-14

**Authors:** Nianshi Wang, Jing Xu, Meiyan Ma, Linghan Shan, Mingli Jiao, Qi Xia, Wanxin Tian, Xiyu Zhang, Limin Liu, Yanhua Hao, Lijun Gao, Qunhong Wu, Ye Li

**Affiliations:** 1 Policy and Management Research Center, School of Health Management, Department of Social Medicine, School of Public Health, Harbin Medical University, No.157 Baojian Road, Nangang District, Harbin, 150086 Heilongjiang China; 2 Heilongjiang Provincial Hospital, 82 Zhongshan Road, Xiangfang District, Harbin, 150086 Heilongjiang China; 3 The Second Affiliated Hospital of Harbin Medical University, 246 Xuefu Road, Nangang District, Harbin, 150001 Heilongjiang China

**Keywords:** Health poverty alleviation, New rural cooperative medical system, Vulnerable population, Middle-aged and elderly people, China

## Abstract

**Background:**

In light of the health poverty alleviation policy, we explore whether the New Rural Cooperative Medical System (NRCMS) has effectively reduced the economic burden of medical expenses on rural middle-aged and elderly people and other impoverished vulnerable groups. The study aims to provide evidence that can be used to improve the medical insurance system.

**Methods:**

Data were obtained from the 2015 China Health and Retirement Longitudinal Study (CHARLS). The method of calculating the catastrophic health expenditure (CHE) and impoverishment by medical expense (IME) was adopted from the World Health Organization (WHO). The treatment effect model was used to identify the determinants of CHE for rural middle-aged and elderly people.

**Results:**

The incidence of CHE in rural China for middle-aged and elderly people is 21.8%, and the IME is 8.0%. The households that had enrolled in the NRCMS suffered higher CHE (21.9%) and IME (8.0%), than those that had not enrolled (CHE: 20.6% and IME: 7.7%). The NRCMS did not provide sufficient economic protection from CHE for households with three or more chronic diseases, inpatients, or households with members aged over 65 years. Key risk factors for the CHE included education levels, households with inpatients, households with members aged over 65 years, and households with disabilities.

**Conclusions:**

Although the NRCMS has reduced barriers to the usage of household health services by reducing people’s out-of-pocket payments, it has not effectively reduced the risk of these households falling into poverty. Our research identifies the characteristics of vulnerable groups that the NRCMS does not provide enough support for, and which puts them at a greater risk of falling into poverty due to health impoverishment.

## Introduction

Poverty is a silent war. According to the World Bank’s international poverty line, whose threshold stands at $1.90 US dollars a day, 10% of the total world population in 2015 was poor [1]. Post 2007, 75.9% of the poor have been mainly concentrated in middle-income countries [2]. Poverty has been a common challenge for all countries. In 2013, one of the 17 goals of sustainable development, proposed in the UN’s “The 2030 Agenda for Sustainable Development,” was to end all forms of poverty by expanding social protection by 2030, which would further support global sustainability [3].

China’s population of rural poor had decreased from 770 million in 1978 to 30.46 million at the end of 2017, and the total number of people lifted out of poverty in the country was about 740 million [4]. About 19 million people are lifted out of poverty each year. China’s contribution alone, to total global poverty reduction, exceeds 70% [5]. Before the reform and opening-up, China’s rural poverty rate was as high as 97%, which means only 3% of the rural population was not poor. However, post the reform and opening-up in 1978, China’s rural poverty rate was reduced to 3.1% by the end of 2017 [4].

**Fig. 1:**
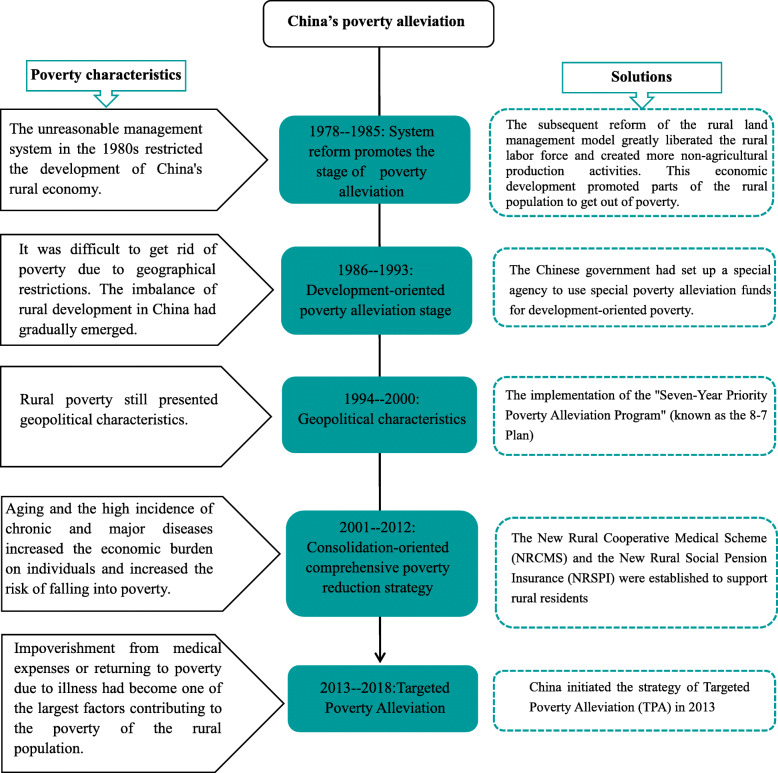
China's poverty alleviation

China’s exploration of poverty alleviation in rural areas has gone through five stages since 1978 (Fig. 1). In the first stage from 1978 to 1985, considering the current poverty standards, the rural population living in poverty decreased from 770 million to 660 million, with the incidence of IME (impoverishment by medical expense) falling to 78.3% [4]. At this stage, the average annual poverty reduction rate in rural areas was 2%. In the second stage, from 1986 to 1993, the proportion of poor people in the total rural population fell from 14.8 to 8.7% [6]. Through economic development, the isolated, poverty-stricken areas, in central and western regions, were lifted out of poverty, [7] and the annual poverty alleviation rate reached 6.10%.

The third stage, from 1994 to 2000, was when the National Plan for Poverty Reduction was introduced, China’s rural poverty alleviation work entered a stage of rapid acceleration. In the 3 years from 1997 to 1999, every year about 8 million people were moved above the poverty line by solving for the lack of food and clothing and through that, alleviating poverty [6]. China’s rural areas witnessed the highest poverty alleviation since the 1990s. The resulting rapid economic growth pushed the annual average poverty alleviation rate in the rural areas up to 9.02%. In the fourth stage from 2001 to 2012, poverty-reduction faced new challenges [8]. In 2012, the population of the poor in Chinese rural areas had decreased to 98.99 million, and the incidence of IME had fallen to 10.2% [4].

In the 2001–2012 period, the economy continued to develop at a relatively fast pace in, but the annual average poverty reduction rate dropped from 9.02 to 7.1%. The trickle-down effect of economic development was not enough to lift the remaining population out of poverty. The marginal diminishing effect of existing antipoverty policies and the changes in the characteristics of the poverty-ridden population, posed great challenges to poverty alleviation that could not be effectively solved by existing strategies [9]. In order to achieve the goal of completely eradicating poverty by 2020, China’s poverty alleviation strategies had to change with the times [10]. Therefore, in the fifth stage, China implemented the strategy of Targeted Poverty Alleviation (TPA) in 2013, which was important to innovate and develop the poverty alleviation methods [11]. Under this policy, the annual average poverty reduction rate increased to 15.97% during the 2013–2018 period.

However, the long-standing urban-rural dualistic structure, along with the natural resources and social factors that restrict rural development, resulted in a total of 43.4 million people still living in poverty at the end of 2016 [4]. Although the five-guarantee households enjoy the policy assistance provided by the national policies for their food, clothing, housing, medical care and burial, such households are inherently vulnerable to poverty due to the elderly and disabled members [12].At the same time, according to the statistical bulletin issued by the National Bureau of Statistics of China, among the poor people in rural China, the number of people in poverty due to an illness was 44.1%, and those in poverty due to long-term chronic diseases (such as cardiovascular diseases) was 22.8% [13]. The rural population had a serious economic burden. Impoverishment from medical expenses or returning to poverty due to illness, had become one of the largest factors contributing to the poverty of the rural population. It is estimated that the annual growth rate of medical expenses for the elderly in China will increase to 2.2% in 2010–2030, which is much higher than that of the United States and other Organization for Economic Cooperation and Development (OECD) countries (0.3–0.5%) [14]. The number of poor people in rural China is three times more than those in urban areas [15]. At the end of 2017, China's national population reached 1.39 billion, of which the population aged over 60, reached 240.9 million, accounting for about 17.3% of the total national population [16]. Moreover, the elderly have always been susceptible to chronic diseases. The economic burden of diseases caused by chronic illnesses accounts for 70% of the economic burden of all diseases [17]. Older people with chronic diseases must bear the accompanying economic burden of the disease throughout their lives [18]. As the most direct and effective means of health poverty alleviation, the medical insurance system aims to not only protect the health of residents, but also to help them avoid the economic risks associated with the use of medical services [19].

In 2017, the coverage rate of the New Rural Cooperative Medical System (NRCMS) was more than 98% [20]. However, the disadvantages of the NRCMS have been gradually exposed. While the NRCMS reduced the threshold for the rural population to avail medical services, it also increased the economic burden resulting from diseases. In 2016, the hospitalization reimbursement rate of Urban Employee Medical Insurance was 75%, which was 20% higher than the NRCMS reimbursement rate [21]. Although the coverage of NRCMS was the highest among the three basic medical insurance programs, it lagged behind the other two programs both in terms of depth and height of coverage [21]. Changes in the demographic structure have led to changes in household demand. Can health poverty alleviation accurately target the most vulnerable segments of the population? Has the NRCMS really alleviated the economic burden of the rural population? Has the overall health poverty alleviation goal been reached? These questions deserve a definitive answer. Therefore, from a multi-dimensional perspective, we scanned the characteristics of poverty-stricken groups over 45 years of age in rural areas, and identified the key challenges that led to the failure of the NRCMS.

## Method

### Data source and sampling method

This study used the China Health and Retirement Longitudinal Study (CHARLS) database, which is a large-scale interdisciplinary survey project jointly conducted by the National Development Research Institute and the Social Science Research Center of Peking University. In 2015, using multi-stage sampling and Probabilities Proportional to Size (PPS) sampling methods, CHARLS randomly selected 45-year-olds from the survey households that were, in turn, selected from 450 communities in 150 counties of 28 provinces (autonomous regions and municipalities) across China. After data cleaning (eliminating abnormal and incomplete data), 7080 households and a 13,740 people remained and were used to calculate the catastrophic health expenditure (CHE). This database will be openly and freely available to academia 1 year after the survey is completed.

### Statistical analysis

#### Catastrophic health expenditure calculation

The method recommended by the World Health Organization (**WHO**) was used to calculate the CHE. The key variables in the algorithm included, Out-of-pocket health expenditure (**OOP**) which is the amount paid by a household member in cash when purchasing health care services, household consumption expenditure (**EXP**) which is the currency and goods used in all goods and services consumed by the household and a household’s capacity to pay (**CTP**), which is the non-subsistence spending of a household as a share of total household consumption expenditure.

When OOP exceeds 40% of the household’s ability to pay, the household is considered to have catastrophic health expenditures [22].

#### Treatment-effect model and instrumental variables

Since the relationship between medical insurance and catastrophic health care expenditures has a joint causality, and taking into account the literature review, we assumed that householder participation in medical insurance was an endogenous variable [23–27]. To further explore whether it was truly endogenous, we used the DWH (Durbin-Wu-Hausman) test (because data had heteroscedasticity *P* = 0.000 < 0.05) which showed *P* = 0.0493 < 0.05, indicating that householder participation in medical insurance was indeed endogenous. In order to address this endogeneity, we applied instrumental variables (IV) with treatment-effect models [28].

The basic idea was to identify an instrumental variable that was related to the endogenous variables (whether the householder was insured), but not related to the residual term of the outcome variable (CHE) [29]. Based on previous literature, the regional medical insurance participation rate and provinces were selected as potential instrumental variables. We further screened for strong instrumental variables through three steps: a) The over-identification test was used to confirm that the variable was exogenous and not related to the residual term of the outcome variable (CHE); b) The F-test was conducted to identify the correlation between instrumental variables and endogenous variables (whether the householder was insured); and c) The redundancy test was adopted to check if tool variables were redundant. Finally, the “regional medical insurance participation rate” was identified as the effective instrument variable to enter the treatment-effect model. (Please see the detailed results in Supplementary Table 1.)

## Results

### Sample characteristics

The total sample comprised 7080 households and 13,740 individuals, the final participation rate of the sample population was 85.8%. (Table 1) 53.1% of household heads were male, and about 32% of households had members aged over 65 years. 66.5%, of the total population had primary and junior high school education and more than half (62.5%) of middle-aged and elderly people had chronic diseases.

**Table 1:** Selection and description of variables

Variable	Variable Value	Percentage (%)
Outcome variable	0 = Catastrophic health expenditure did not occur	78.2
	1 = Catastrophic health expenditure occurred	21.8
Endogenous variable	0 = Uninsured	14.2
	1 = Insured	85.8
Gender of household head	1 = Male	53.1
	2 = Female	46.9
Educational level of household head	1 = Illiterate	24.7
	2 = Primary and junior high school	66.5
	3 = High school	8.6
	4 = University and above	0.1
Career of household head	1 = Agriculture	54.1
	2 = Industry	20.9
	3 = Unemployed	19.5
	4 = Retired	5.5
Households with members aged over 65 years	1 = Yes	32.7
	2 = No	67.3
Households with disabled member	0 = No	66.9
	1 = Yes	33.1
Households with inpatients	1 = Yes	12.5
	2 = No	87.5
Household members with chronic disease	0 = 0 species	37.5
	1 = 1 species	35.3
	2 = 2 species	20.0
	3 = 3 species or more	7.2
Family size	1 = 1	61.8
	2 = 2	35.7
	3 = 3	2.4
	4 = ≧4	0.1
Region	1 = East	34.7
	2 = Central	42.0
	3 = West	23.3

### The overall prevalence, utilization of health services

Among households with chronic diseases (Table 2), the prevalence rate of households with three chronic diseases was the highest (17.2%(*p* = 0.02)). The higher demand for medical services meant higher health service utilization, with the highest inpatient rate of 15.1% (*p* = 0.005) being for households with three chronic diseases. However, their inpatientl reimbursement rate was only 45.8%, nearly 7% lower than that of households with two chronic diseases. Households with members aged over 65 years had higher health service utilization and high demand for medical services, and a inpatient reimbursement rate of 53.7% (*p* = 0.001), which was 1.3 times that of households without members aged over 65 years. Households with disabled members also showed the same trend. Meanwhile, the prevalence rate of disease decreased with an increase in the educational level. Compared with households with a high school education, households who were illiterate showed a higher utilization of health services, inpatient rate (14.3%), and outpatient rate (20.6%). Compared with the central region, the health demand and utilization in the western region were both higher, with 20.6% (*p* = 0.000) prevalence and 16.4% (*p* = 0.000) inpatient rate, respectively. Meanwhile, the western region had the highest reimbursement rate of the three regions. The prevalence of uninsured households (15.9%) was higher than that of insured households (14.8%); however, both outpatient and inpatient rates were lower for the uninsured households compared with insured households, while the payment ability of the two was almost the same.

**Table 2:** Health-care needs and service utilization

Variables	Variable value	Prevalence(%)	Inpatient rate (%)	Outpatient rate (%)	Inpatient reimbursement ratio (%)
Whether to participate in insurance	Insured	14.8	13.2	20.4**	45.5
	Uninsured	15.9	12.5	16.6	/
Households with members aged over 65 years	Yes	16.4**	15.3**	21.0	53.7**
	No	14.2	12.1	19.6	39.9
Species of chronic diseases	0 species	13.4**	11.2**	18.8	43.0
	1 species	15.60	13.8	20.8	45.1
	2 species	16.0	14.9	21.0	52.7
	3 species	17.2	15.1	19.6	45.8
Educational level	Illiterate	16.0	14.3	20.6	44.5**
	Elementary school and junior high school	14.8	13.1	20.2	44.6
	High school	13.0	12.1	19.2	45.2
	University and above	12.5	22.2	11.1	30.5
Family size	1	15.1	13.4	19.7	47.9
	2	15.1	12.5	20.2	42.8
	3	13.9	14.0	22.0	43.5
	4	/	25	25	/
Disabled	Yes	17.2**	14.9**	20.5	47.8
	No	13.8	12.2	19.7	45.2
Inpatient	Yes	23.8**	100**	34.9**	46.2
	No	13.9	/	17.8	/
Region	East	10.6**	10.7**	18.0**	44.3
	Central	15.6	13.2	19.9	44.6
	West	20.6	16.4	23.2	50.4

*P < 0.05; **P < 0.01

### Catastrophic health expenditure (CHE) and impoverished by medical expenses (IME) in different households

The above data (Table 3) showed that the highest incidence of CHE was concentrated in households with members who have three chronic diseases (38.0% (*p* = 0.000)), inpatients members (31.0%(*p* = 0.000)), or households with members aged over 65 years (30.5% (*p* = 0.000)). The NRCMS is an insufficient reimbursement for households that have various chronic diseases, resulting in the highest incidence of CHE, and the households with members aged over 65 years were more at risk of impoverishment by medical expense (*p* = 0.000). The incidence of IME (11.1%) was 4.7% higher in the households with members aged over 65 years, than those without members aged over 65 years. The capacity to pay increased with the increase in the education level (*p* = 0.000). The lower capacity to pay of the illiterate group leads to the higher incidence of IME (10.3%) which is 4.3% higher than the IME of the households with high school education (*p* = 0.004).

**Table 3:** Catastrophic health expenditure and impoverished by medical expenses in different households

Variables	Variable value	Out-of-pocket/capacity to pay(%)	Incidence of catastrophic health expenditures(%)	Incidence of impoverishment by medical expense(%)
Participate in insurance	Insured	19.0	21.9	8.0
	Uninsured	19.9	20.6	7.7
Households with members aged over 65 years	Yes	25.9**	30.5**	11.1**
	No	16.9	17.6	6.4
Species of chronic diseases	0 species	15.7**	16.1**	5.6**
	1 species	19.3	21.7	7.6
	2 species	23.2	26.5	10.6
	3 species	34.1	38.0	14.8
Educational level	Illiterate	21.5**	26.2**	10.3**
	Elementary school and junior high school	20.3	21.6	7.7
	High school	14.9	17.0	6.0
	University and above	5.6	0.0	0.0
Family size	1	23.0*	24.6**	9.0**
	2	15.3	17.1	6.3
	3	16.1	16.5	6.1
	4	38.7	25	/
Disabled	Yes	26.0**	27.3**	10.2**
	No	16.7	19.0	6.9
Inpatient	Yes	29.7**	31.0**	11.0**
	No	17.6	20.4	7.6
Region	East	17.8	20.8	7.8
	Central	20.8	22.6	8.4
	West	18.5	21.5	7.5

*P < 0.05; **P < 0.01

Among the different regions, the incidence of CHE in the Eastern, central and Western regions exceeded 20% and the Central region showed the highest CHE at 22.6%. Surprisingly, the incidence of CHE for insured households (21.9%) was higher by 1.3% than the CHE for uninsured households, and the incidence of IME was also higher, at 8.0%.

### Prevalence rate and utilization of health service demand under different insurance conditions

Since the incidences of CHE and IME in the insured households were higher than that in the uninsured households, we analyzed the health needs, utilization, and OOP payment of different insured households using different characteristic dimensions and uncovered two results as seen in Table 4.

**Table 4:** Utilization of health service under different insurance conditions

	Insured				Uninsured			
Variables	Prevalence rate (%)	Inpatient rate (%)	Outpatient rate (%)	Out-of-pocket (yuan)	Prevalence rate (%)	Inpatient rate (%)	Outpatient rate (%)	Out-of-pocket (yuan)
Family size 1	14.8	13.5*	20.1	310.46	16.9	12.3	16.2	330.35
Family size 2	15	12.4	20.5	332.16	16.2	13.3	17.7	421.5
Family size 3	14.6	14.2	22.8	343.22	8	11.8	15.7	392.24
Family size 4	/	33.3	33.3	138	/	/	/	417
Households with members aged over 65 years	16.6**	15.3**	21.6*	321.13**	14.7	15.5	17.1	368.12
Households without members aged over 65 years	14	12.2	20	323.00	16.3	10.8	16.8	349.33
Having No chronic disease	13.11	11.1	19.1	306.69	14.8	11.7	16.2	319.49
Having One chronic disease	15.7**	14.8**	21.3	322.50	16.6	13.5	17.6	342.64
Having Two chronic diseases	15.8	13.8	21.6	343.78	16.7	13.8	17.6	500.61
Having Three chronic diseases	16.9	15.6	20.5	409.75	17.6	9.5	11.7	310.44
No disability	13.6	12.3	20.2*	304.34	15	11.4	15.4	352.03
Disability	17.1**	14.9**	20.8*	349.58**	17.7	14.7	18.8	391.15
Inpatient	23.4**	100**	34.5**	536.26**	27.3	100**	38.6**	467.06
No Inpatient	13.8	0	18.3	289.03	14.7	0	13.5	354.54
Illiteracy	15.5	13.1	20.2	276.49	16	17	16.5	406.03
Elementary school and junior high school	14.6	13.4	20.6	317.94	16.8	10.3	16.2	356.1
High school	13.1	12.1	19.8	367.78	12.7	11.4	14.6	412.58
University and above	12.5	22.2	11.1	115.47	/	/	/	312.5
The poorest level	14.6	13.3	20.7	58.38**	17.6	10.8	12.9	54.09**
Sub poverty level	14.8	13.1	19.7	129.18	15.6	11.3	17.9	121.97
General level	14.5	12.4	20.7	216.97	14.9	11.5	14.3	192.09
Richer level	15.5	12.4	19.8	393.53	17.7	13	17	369.3
Most wealthy level	14.8	15.3	21.3	1131.39	13.6	17.6	22.9	1158.42

*P < 0.05; **P < 0.01

First, the results showed that, for households with three chronic diseases, inpatient, and below-average household economic level, health insurance increased the residents’ use of health services while also increasing the amount of OOP payment. Among households with chronic diseases, the prevalence of the insured households (15.7–16.9%) (*p* = 0.006) was lower than that of the uninsured households (16.6–17.6%). Households with three chronic diseases and who were insured had the highest inpatient rate (15.6%) (*p* = 0.002), which was 6.1% higher than that of households that were uninsured. Although insurance coverage increased the utilization of health services, the OOP payment for insured households with three chronic diseases, was 1.3 times that of the OOP payment for uninsured households. However, in other households with one or two chronic diseases, the OOP payments for insured households were much lower than that of uninsured households. For the households with an average or below-average economic level, having insurance significantly improved their utilization of medical services. The medical service utilization rate of outpatients in the poorest insured households (20.7%) is 7.8% higher than that of outpatients in the poorest uninsured households. However, the OOP payment (58.38–216.97 yuan) of insured households was generally higher than that of uninsured households (54.09–192.09 yuan) (*p* = 0.000).

Second, for households with disabilities, an education level below primary, and a family size with two members or less, having insurance increased the utilization of health services, but their OOP payment did not increase. Among the households with disabled people, the prevalence of households with insurance (17.1%) (*p* = 0.000) was lower than that of households without insurance (17.7%). Their inpatient and outpatient utilization rates were higher than those of the latter. The outpatient utilization rate (20.8%) in particular was 2% higher than the rate of the uninsured households. Having insurance reduced the threshold of health service utilization and provided corresponding economic protection with the OOP being less than the OOP of uninsured households at 41.57 yuan. It can be seen that, in families with two members or less, being covered by the NRCMS ensured that insured households with lowered health demand had higher utilizations, with the outpatient rate (20.1%) 3.9% higher than that of uninsured households. Households with lower educational levels also showed the same trend. Looking at health service utilization, the OOP payment of the illiterate population (276.49 yuan) who had insurance was far lower than the 406.03 yuan OOP payment of uninsured households. Among the wealthiest groups, although the OOP payment of uninsured households was 1158 yuan, higher than that of the insured households, their utilization of health services was also higher. The inpatient rate of the wealthiest and uninsured households was 17.6%, higher than that of the insured households, which showed that medical expenses increased with the increased availability of health services. Therefore, the choice of going for insurance had little effect on the wealthiest households.

### Catastrophic health expenditure (CHE) and impoverished by medical expenses (IME) under different insurance schemes

**Fig. 2:**
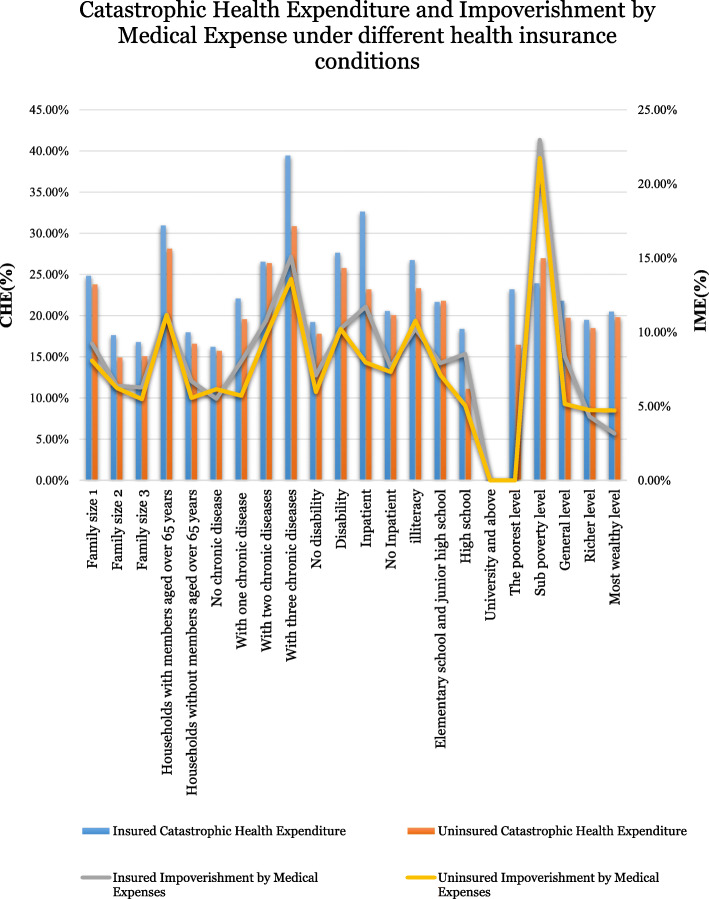
Catastrophic health expenditures and Impoverishment by Medical Expenses under different health insurance schemes

Considering different insurance schemes (Fig. 2), except for the sub-poor, no chronic disease and most wealthy level households, regardless of the incidence of CHE or IME, the insured households had higher CHE and IME rates than the uninsured households. The most at-risk households were the insured households with three types of chronic diseases (39.44%), inpatients (32.64%), and members aged over 65 years (30.9%). Among the households with three chronic diseases, the incidence of CHE for those enrolled in medical insurance was 8.58% higher than the incidence for the uninsured. In households with inpatients, the incidence of CHE for households enrolled in medical insurance was 1.4 times that of the uninsured. In households with members aged over 65 years, the incidence of CHE for households enrolled in medical insurance was 2.82 % higher than the uninsured households

However, in households with members aged over 65 years but without medical insurance, the incidence of CHE was still 1.2 times that of the households with two chronic diseases, enrolled in medical insurance. Further, for the sub-poor families, regardless of whether they had medical insurance, the incidence of IME was high (22.97% for insured households and 22.74% for uninsured households). However, it is worth noting that the incidence of CHE in the sub-poor households was not the highest (23.93/26.96%). The second highest incidence of IME was in households with three chronic diseases, 15.08% in insured households and 13.58% in uninsured households. Finally, 11.71% of the insured households with inpatients had an IME 1.4 times higher than that of households with inpatients (7.95%) without insurance. If the household head had a university level education, the incidence of CHE and IME were both zero. It can be clearly seen that both the CHE and IME decreased with the increase in the degree of education.

### The result of treatment-effect model

From the results of the treatment-effect model (Table 5), we can see that the education level of the head of the household, households with inpatients, households with members aged over 65 years, disabilities, family size, disease types, as well as whether households were enrolled in insurance, are all determinants of catastrophic household expenditure (CHE) for the middle-aged and elderly people in this study. Among them, households with hospitalization, households with members aged over 65 years, disabled members, and those with chronic disease are the most significant factors impacting CHE. In rural China, income seemed to have no significant association with the incidence of CHE and IME for middle-aged and elderly people.

**Table 5:** The result of treatment-effect model

Variables	Coefficient	SE	Z	*P*
Household head gender	0.0044	0.0137	0.32	0.749
Household head marital status	−0.0003	0.0183	−0.02	0.983
Household revenue logarithm	0.0124	0.0160	0.78	0.431
Household head educational level	−0.0305	0.0124	−2.45	0.015
Inpatient	−0.0722	0.0350	−2.00	0.039
Frequency of inpatient	0.0435	0.0217	2.01	0.045
Region	−0.0119	0.0086	−1.38	0.168
Type of jobs	0.0045	0.0032	1.40	0.160
Households with members aged over 65 years	−0.0802	0.0145	−5.50	0.000
Family size	−0.0379	0.0120	−3.15	0.002
Type of chronic diseases	0.0553	0.0072	7.65	0.000
Disabled	0.0450	0.0141	3.19	0.001
Participate in insurance	0.1558	0.0686	2.27	0.023

The CHE decreased by 3.05% with an increase in education level. The increase in education level had a protective effect on the household. Inpatients in the household increased the incidence of CHE by 7.22%. Households with members aged over 65 years showed an increased risk of CHE by 8.02%. The larger the family size was, the less likely the family was to incur CHE; the increase in family size reduced the incidence of CHE by 3.79%. Disability increased the incidence of CHE by 4.5%. However, being enrolled in insurance increased the risk of CHE, and the CHE of the insured households increased by 15.58% when compared with the CHE of uninsured households.

## Discussion

In 2010, more than 808.4 million people incurred catastrophic health expenditures worldwide, representing 11.7% of the global population [30]. Currently, the overall incidence of CHE among middle-aged and elderly in rural China is 21.8%, and the IME is 8.0%. Among them, the incidence of CHE in households with three chronic diseases is 38.09%, and the rate of IME is 14.84%, which is higher than the rates in China’s urban areas [31] and far exceeds the rate of CHE and IME in other developing countries [32, 33]. Our research found that the IME rate of insured households was much higher than that of uninsured households, and the highest rate was that of the sub-poor households, at 22.97%. The NRCMS reduced barriers to the use of the health services, thereby decreasing households’ OOP payment, affecting households with disabled members, households with elementary and junior high level education, and the households with two or less people. However, in some respects, it was still not efficient in reducing the overall risk of households falling into poverty [34]. We, thus, diagnosed the critical reasons for the failure of the NRCMS:

### Inadequate coverage of households with high demand for health services

First, the reimbursement rate of the NRCMS for the households with high-demand and high-utilization of health services is not adequate, especially for households with three chronic diseases and low education levels. A variety of chronic illnesses and hospitalizations create high demand for medical services, which leads to higher utilization of those medical services and the a weaker ability to pay when compared with other households. Although the NRCMS has lowered the threshold for the utilization of health services, without effective control of medical expenses, it has increased the economic burden on households with three chronic diseases, members aged over 65 years, and inpatients, thus increasing their risk of falling into poverty. This also means that the NRCMS has only increased coverage but has been relatively inadequate in its design for cost reimbursement and benefit packages. Shi’s study noted that the increase in NRCMS coverage was accompanied by an increase in utilization of inpatient services. While reimbursement rates also increased at the same time, the total medical expenses and OOP rose more rapidly [35].

NRCMS has achieved better protection for households with one or two chronic diseases, but the protection is still insufficient in the households where there was a superimposition of various chronic diseases. The incidence rate of IME in the superimposed households was 2.64 times higher than in the households without chronic diseases. Combined with the physiological vulnerability of the middle-aged and the elderly and the risk of multiple chronic diseases, the insufficient tilt system design of NRCMS has increased the risk of these vulnerable groups to CHE. As a study showed, households with older members and those with chronic illnesses face an increase in demand for the health care system and health expenditures [36]. Moreover, studies had also shown that households with such inadequate protection are more likely to be dragged into poverty due to high expenditures on health care [37, 38].

To truly provide economic protection via the NRCMS for rural middle-aged and elderly people in China, it is necessary to extend the policy coverage to chronic diseases, hospitalized patients, and high-risk households with members aged over 65 years, by increasing the reimbursement rate and expanding the coverage of benefit packages. The benefit packages should not only consider direct medical expenses but also the compensation for indirect medical expenses such as time loss due to long-term care required for chronic diseases.

### Insufficient inclination towards low socio-economic households

Secondly, the design of the NRCMS is not inclined toward the lower socio-economic groups, which is one of the reasons for the impoverishment of the disabled or illiterate households, who have a lower capacity to pay. With the increase in education level, the households’ capacity to pay also improves, leading to a lower incidence of CHE and IME. There was no CHE and IME observed in households with an educational level of university and above. This shows the great protective effect of education on poverty. Our study is largely consistent with previous studies, which indicate that households with lower literacy levels are at greater risk of catastrophic health care expenditures than those with higher literacy levels [32].

Compared to households without disability households, those with disability have a weaker capacity to pay are more likely to incur CHE. Moreover, the incidence of CHE in households with disabilities is higher than that of uninsured households. At least 500 million of the world’s 650 million people with disabilities, are the poorest [39]. Disability is a complex situation that affects not only an individual but also their household, and it reduces the income available to individuals and households [40]. Both the risk of IME and CHE decrease as the family size increases. Some households with fewer members are limited in their ability to access quality and effective health resources and social security benefits. A series of studies has confirmed that larger households mean more working members entering the labor market and more income, which in turn reduces the risk of the household falling into poverty. Once the householder is unemployed, the ability to resist the economic risks of disease will decrease, resulting in IME [37, 41].

### Precise identification and targeting of the characteristics of the poor

Based on our summary of the key reasons of the failure of the NRCMS, it can be seen that that the crux of insurance failures lies in the problem of identifying and targeting the poor. The NRCMS did not accurately identify and assess the characteristics of the poor and incorporate the same into the system design. As an important area under China’s precision poverty alleviation, health poverty alleviation constitutes an institutional means to promote health and poverty alleviation through the integration of basic medical insurance, major medical insurance, medical assistance, and bottom-up protection. However, health alleviation only builds on the original definition of poor population and ignores other potentially poverty-stricken people. Measuring poverty based on income seems to be the most common way, but based on the above research, it is found that the rural poor are not the only ones economically disadvantaged. Those with high demand for health services, households with low economic and educational factors, and low risk of mutual aid, can push “un-poor” households to poverty because of the cost of medical care. Accurate targeting of the poor is of considerable significance for the NRCMS to maximize the effectiveness of health poverty alleviation. While more precise targeting of poor households vulnerable to poverty and the high medical costs associated with multiple chronic disease is needed, we argue that policymakers, who lead process of reforming health insurance, should also focus on increasing reimbursement gradually and ensuring better compensation for chronic diseases.

## Conclusions

Health poverty alleviation is a comprehensive strategy that requires the convergence of different medical systems. A key point is to accurately capture the characteristics of the poor. Our research identified and targeted the characteristics of vulnerable groups using multi-dimensional analysis to try and provide direction for accurate poverty alleviation.

As the basic medical insurance, the NRCMS must accurately and comprehensively identify the characteristics of the poor in the early stage, comprehensively cover all the vulnerable people who are at risk due to diseases, and minimize the economic burden of the disease in the basic medical insurance stage. This can reduce the risk of people living in poverty due to illness. For those who are in poverty and cannot afford high medical expenses, further in-depth protection is provided through major medical insurance and medical assistance. Medical assistance has developed a general framework for identifying the poor, that is, the economically disadvantaged, those with special needs, and the middle-aged and elderly people. However, for middle-aged and elderly people in rural areas above 45 years of age, these high-risks overlap. Thus, medical assistance still lacks a more detailed and accurate definition of poverty. The existing medical security system is only concerned with people with low-income, special hardships, and major diseases. The definition of vulnerable people is only measured from a single economic dimension, but some people in society do not meet the basic conditions for medical assistance. However, this group of people cannot afford to suffer from chronic and major diseases.

Therefore, we must consider not only poor households and households that fall into the “five guarantees,” but also focus on households that are at risk of poverty due to age, education level, family size, chronic diseases, disabilities, and hospitalizations. Based on the above research data, illiterate people, households with three chronic diseases, households with members aged over 65 years, inpatients, and households with small family size should be included in the policy of the Chinese rural middle-aged and elderly medical assistance system.

## Supplementary information

**Additional file 1: Supplementary Table 1.** Result of validity test and correlation test.

## Data Availability

Database available from the CHARLS repository, http://charls.pku.edu.cn.

## Abbreviations

CHE: Catastrophic Health Expenditure
IME: Impoverishment by Medical Expenses
NRCMS: New Rural Cooperative Medical System
NRSPI: New Rural Social Pension Insurance
TPA: Targeted Poverty Alleviation
OECD: Organisation for Economic Cooperation and Development
CTP: A household’s Capacity To Pay
OOP: Out-Of-Pocket health expenditure
EXP: Household Consumption Expenditure
