# Supplementary material for: Targeting vulnerable groups of health poverty alleviation in rural China— what is the role of the New Rural Cooperative Medical Scheme for the middle age and elderly population?
Source: Int J Equity Health. 2020 Sep 14;19:161. doi: 10.1186/s12939-020-01236-x (PMC7489030; doi:10.1186/s12939-020-01236-x)
Supplement: Supplementary file 1 — Additional file 1: Supplementary Table 1. Result of validity test and correlation test. [file 12939_2020_1236_MOESM1_ESM.docx]

Supplementary Table 1 Result of validity test and correlation test

| Instrumental  variables | DWH Test | Over-identification  Test | F-Test | Redundancy Test |
| --- | --- | --- | --- | --- |
| participation rate | chi2(1)=3.88075  (p=0.0493<0.05)* | chi2(1)=0.0311  (p=0.86>0.05)* | F=138>10 | Chi-sq(1) =0.0000** |
| provinces |  |  |  | Chi-sq(1) =0.9071 |

*P<0.05; **P<0.01.
